# Combinations of genetic variants associated with bipolar disorder

**DOI:** 10.1371/journal.pone.0189739

**Published:** 2017-12-21

**Authors:** Erling Mellerup, Ole A. Andreassen, Bente Bennike, Henrik Dam, Srdjan Djurovic, Martin Balslev Jorgensen, Lars Vedel Kessing, Pernille Koefoed, Ingrid Melle, Ole Mors, Gert Lykke Moeller

**Affiliations:** 1 Laboratory of Neuropsychiatry, Department of Neuroscience and Pharmacology, University of Copenhagen, Copenhagen, Denmark; 2 Department of Psychiatry, Oslo University Hospital and Institute of Psychiatry, University of Oslo, Oslo, Norway; 3 Psychiatric Centre Copenhagen, Department O, Copenhagen University Hospital, Rigshospitalet, Copenhagen, Denmark; 4 Department of Medical Genetics, Oslo University Hospital and Institute of Psychiatry, University of Oslo, Oslo, Norway; 5 Centre for Psyciatric Research, Aarhus University Hospital, Skovagervej 2, Risskov, Denmark; 6 Genokey ApS, ScionDTU, Technical University Denmark, Agern Allé 3, Hoersholm, Denmark; Universidade Nova de Lisboa Instituto de Higiene e Medicina Tropical, PORTUGAL

## Abstract

The main objective of the study was to find genetic variants that in combination are significantly associated with bipolar disorder. In previous studies of bipolar disorder, combinations of three and four single nucleotide polymorphisms (SNP) genotypes taken from 803 SNPs were analyzed, and five clusters of combinations were found to be significantly associated with bipolar disorder. In the present study, combinations of ten SNP genotypes taken from the same 803 SNPs were analyzed, and one cluster of combinations was found to be significantly associated with bipolar disorder. Combinations from the new cluster and from the five previous clusters were identified in the genomes of 266 or 44% of the 607 patients in the study whereas none of the 1355 control participants had any of these combinations in their genome.The SNP genotypes in the smaller combinations were the normal homozygote, heterozygote or variant homozygote. In the combinations containing 10 SNP genotypes almost all the genotypes were the normal homozygote. Such a finding may indicate that accumulation in the genome of combinations containing few SNP genotypes may be a risk factor for bipolar disorder when those combinations contain relatively many rare SNP genotypes, whereas combinations need to contain many SNP genotypes to be a risk factor when most of the SNP genotypes are the normal homozygote.

## Introduction

This study is the third study using a material of 607 bipolar patients and 1355 controls from Denmark and Norway in which 803 SNPs in 55 genes were analyzed.

In bipolar disorder hyperactivity is the main symptom of the manic phase, possibly reflecting faster signal transmission in the brain. Based on this assumption we have investigated genes related to the action potential, refractory period, ion channels and CNS myelination. Among such genes 55 were selected based on a search in Medline for genes associated with bipolar disorder. In the first study of this material a table shows the 55 genes and the corresponding proteins [1].

The aim of the three studies has been to analyse combinations of SNP genotypes taken from the 803 SNPs. In the first study combinations of three SNP genotypes were analysed [1]. The theoretical number of such combinations taken from 803 SNPs is 2,3x10^9^ and at that time our methodology did not allow analysis of larger combinations. Four clusters of combinations were found to be significantly associated to the disorder and 26% of the patients had combinations from the clusters in their genome, in contrast to 0% of the controls. In the second study technological improvement allowed analysis of combinations of four SNP genotypes [2]. The theoretical number of such combinations taken from 803 SNPs is 1,4x10^12^. One cluster of combinations significantly associated to bipolar disorder was found and a further 8% of the patients had combinations from this cluster in their genome, in contrast to 0% of the controls. In the present study it has been possible to analyse combinations up to 10 SNP genotypes. The theoretical number of combinations of 10 SNP genotypes taken from 803 SNPs is 1,7x10^28^.

In a polygenic disorder one or more combinations of genetic variants are the basis for the disorder, and identification of these combinations may be a major step towards an understanding of the disease aeitiology. In the previous studies [1,2] combinations containing three and four SNP genotypes were analyzed, but disease related combinations may probably also be much larger, hereby justifying the search for large combinations. Larger combinations may be combinations of already identified small combinations, but they may also contain SNP genotypes not present in the small combinations, and may identify additional patients having disease related combinations in their genome.

The number of genetic variants in a combination that is basis for a polygenic disorder is unknown, because such a combination has never been described, partly because it is difficult to identify disease related combinations among the extremely high numbers of combinations. In order to reduce the number of combinations to be analyzed, and to increase the probability of finding disease related combinations, we have used a research strategy where only combinations found exclusively in patients have been selected for statistical analysis [1–5]. This strategy is based on the implicit statement in the concept of polygenic disorders that combinations of genetic variants that constitute or contribute to the basis for a disorder will normally not be found in healthy subjects genetically unrelated to the patients.

Other clinical studies of genetic variant combinations have predominantly investigated associations between two-variant combinations and networks of genetic variants, however, none of these studies have analyzed combinations occurring exclusively in patients [6–13].

## Materials and methods

### Patients and controls

The patient sample consisted of 607 bipolar patients and 1355 controls from Denmark and Norway.

The Norwegian Scientific-Ethical Committees, the Norwegian Data Protection Agency, the Danish Scientific Committees, and the Danish Data Protection Agency approved the study. All patients gave written informed consent prior to inclusion in the project.

### Genes and SNPs

The genes were selected based on the relation of the corresponding functional proteins to various aspects of the action potential, including ion channels, proteins in the nodes of ranvier and proteins involved in CNS myelination, as well as proteins that are targets for mood stabilizing drugs or have been related to bipolar disorder in previous studies. SNP selection was performed at the HapMap website and is described in details in reference [1] which also shows the 55 selected genes, the corresponding proteins and the number of SNPs analysed in each gene.

### Combinations

Studying genetic variant combinations can involve scanning and analyzing data sets containing billions of such combinations. Even relatively powerful computers may be unable to perform such a task. Apart from increased computer power, two technological developments have helped to decrease the scanning time for combinations: massively parallel computing by graphics processing units [14,15], and cloud computing [16,17].

Specialized software is also necessary for analyzing genetic variant combinations. Algorithms and data mining tools have been developed for this purpose, based on methods such as regression analysis, Bayesian statistics, Boolean algebra, and array mathematics [18]. A review lists 27 publicly available applications for analyzing combinations of genetic data [6].

The theoretical number of combinations of *r* SNP genotypes taken from *n* SNPs can be calculated using the formula, *n*!/*r*!(*n* − *r*)!*×3*^*r*^, where *n* represents the number of genetic variants analyzed in a study, and *r* represents the number of genetic variants per combination, and *3*^*r*^ is due to the 3 possible genotypes for each SNP. In the present study *n* is 803 and the highest value of *r* is 10, which means that the theoretical number of combinations of 10 SNP genotypes is 1,7x10^28^.

Ideally the 803 SNPs would be scanned for combinations of two SNP genotypes, then for combinations of three SNP genotypes, and so on layer after layer until combinations of ten SNP genotypes are reached. However, the available tools do not allow such a brute force analysis of all possible combinations, so instead smaller groups of combinations are selected. These combinations are selected by the use of three principles that should increase the chances of the selected combinations being significantly associated to bipolar disorder:

A. Only SNP genotypes and combinations occurring significantly more often in patients than in controls are used to form the next layer of combinations.

B. In each layer of combinations only those that occur exclusively in patients are selected.

C. Among these patient specific combinations only those that are common for many patients are selected.

The number of selected combinations depends on the chosen *p-*value parameter in the test used to analyze the distribution of combinations between patients and controls (A), and on the chosen number of patients having a common combination (C). Using a low *p-*value and a high number of patients sharing a common combination a number of selected combinations can be obtained that is small enough to allow analysis by our methods.

It should be emphasized that this procedure allow us to select combinations of genetic variants associated to bipolar disorder, but most of the combinations in the data set are not selected and thus remain unanalyzed, and it cannot be excluded that some of these may be associated with bipolar disorder.

### Clusters of combinations

The three principles should increase the chances that the selected combinations are significantly associated with the disorder. However, due to the large number of possible combinations most of them may not occur in the material, many may occur once, and few may found in several patients. Accordingly, the number of patients exhibiting the same combination of genetic variants may be too small to confirm a statistically significant association between a single combination and the disorder. In this situation, clusters of the selected combinations can be tested for significant association with the disorder. A cluster is defined as a group of combinations that share at least one common SNP genotype. Patients belonging to a cluster are those who have combinations from that cluster in their genome.

### Statistics

Chi-square tests can be used to determine whether the distribution of a genetic variant combination differs significantly between patients and control subjects. Permutation tests can be employed to assess whether the combinations and clusters found exclusively in patients are significantly associated with bipolar disorder. Thus, the selected combinations in each layer from 2 to 10 SNP genotypes can be organized into a number of clusters that are tested by permutation tests, followed by Benjamini-Hochberg correction for multiple testing, to see if some are significantly associated with bipolar disorder.

Permutation tests can be used to analyze many different genetic variant combinations selected from a data set [19]. Thus, a permutation test can be used to evaluate the assumption that, among genetic variant combinations found exclusively in patients; combinations common to many patients are more likely to be significantly associated with the disorder than combinations found in few patients. In a permutation test, the null hypothesis is that the observed data are exchangeable with respect to groups—in this case, the patients and controls. For this analysis, indices for patients and controls would be randomly re-distributed, creating two new groups of pseudo-patients and pseudo-controls of the same sizes as the original groups. This would be repeated for example, 1,000 times and the combinations found exclusively in pseudo-patients and being common to many pseudo-patients would be identified in each of the 1,000 permutations. The null hypothesis is validated if the number of pseudo-patients having these combinations in their genome is the same or higher than in the original data set in more than 50 of the 1,000 permutations (*p* > 0.05), which would suggest that the combinations found exclusively in patients and also being common to many patients may be random findings.

## Results

Among the 803 SNPs no single SNP genotype and no single combination of the SNP genotypes was found to be significantly associated with bipolar disorder. No clusters of combinations of two SNP genotypes were found to be significantly associated with bipolar disorder. The previous finding of four clusters of combinations of three SNP genotypes [1] and one cluster of combinations of four SNP genotypes [2] significantly associated with bipolar disorder was confirmed in the present study. No clusters of combinations of five, six, seven, eight and nine SNP genotypes were found to be significantly associated with bipolar disorder. One cluster of combinations of 10 SNP genotypes was found to be significantly associated with bipolar disorder. This cluster contains 21 combinations and 31 SNP genotypes. 85 patients belong to the cluster, of which 28 also belongs to the clusters of combinations of three and four SNP genotypes. Only two of the 31 SNP genotypes are also found in the other clusters. The 21 combinations show a very large overlap of SNP genotypes. Almost all the SNP genotypes in the cluster are the normal homozygote, and none are the variant homozygote, which is in contrast to the other three and four SNP genotype clusters.

Table 1 shows a part of the cluster with combinations of 10 SNP genotypes. S1 Table shows the whole cluster.

**Table 1:** Part of the cluster with combinations of 10 SNP genotypes.

SNP1	SNP2	SNP3	SNP4	SNP5	Patients
PDE4B_rs4288570	TNR_rs2021832	NCAM1_rs17115280	SCN8A_rs7963772	CNTN1_rs11178961	41
PDE4B_rs4288570	TNR_rs2021832	SCN8A_rs7963772	MAP2_rs6751280	CNTN1_rs11178961	40
TBR1_rs3769956	TNR_rs2021832	PPP2R2C_rs4440300	NCAM1_rs4646982	SCN8A_rs3741705	40
PDE4B_rs4288570	NCAM1_rs17115280	SCN8A_rs7963772	CNTN1_rs12307865	NFASC_rs7534993	41
PDE4B_rs4288570	NCAM1_rs17115280	SCN8A_rs7963772	CNTN1_rs11178961	NFASC_rs7534993	41

Five of the 10 SNP genotypes in each combination are shown for the first five of the 21 combinations. All SNP genotypes are the normal homozygote, except NFASC_rs7534993 which is the heterozygote.

Of the 85 patients belonging to the cluster 57 did not belong to other clusters. The number of patients belonging to the clusters of combinations of three SNP genotypes was 156 [1], the number of patients belonging to the cluster of combinations of four SNP genotypes was 53 [2], thus 266 or 44% of the 607 patients have combinations in their genome from clusters significantly associated with bipolar disorder in contrast to 0% of the 1355controls.

## Discussion

Working with combinations is a mathematical, computational and statistical challenge even with a sample as small as 803 SNPs. Combinations of three SNP genotypes taken from 803 SNPs could be analyzed brute force [1], but already with combinations of four SNP genotypes this was not possible [2]. The theoretical number of combinations of 10 SNP genotypes taken from 803 SNPs is so high (1,7x10^28)^ that analysis or even a scanning to see how many of these combinations are actually present in the material is impossible with our present tools. Instead samples of combinations are selected that are small enough to be analyzed. The selection of these combinations is based on principles that should increase the chances of obtaining combinations associated with bipolar disorder. As described in Materials and Methods only SNP genotypes and combinations occurring significantly more often in patients than in controls are selected, and among these only those combinations that occur exclusively in patients are selected, and among these only those that are common for many patients are selected.

Despite these narrow selection criteria single combinations of SNP genotypes significantly associated to bipolar disorder could not be found, probably because the number of combinations is so high that most of the combinations only are found once or very few times in the sample, and thus cannot obtain statistical significance. However, combinations showing some similarity as a shared SNP genotype can be grouped into clusters that can be tested statistically.

No clusters of combinations of two SNP genotypes were found to be significantly associated with bipolar disorder. Four clusters of combinations of three SNP genotypes were found to be significantly associated with bipolar disorder. These four clusters were almost identical to the four clusters found previously [1], the only change was that a few SNP genotypes were missing in the clusters found by the selection procedure, compared with the clusters found by brute force analysis of all combinations. One cluster of combination of four SNP genotypes was found to be significantly associated with bipolar disorder, and this cluster was identical with the cluster found previously [2]. Although many clusters of combinations of five, six, seven, eight and nine SNP genotypes were found none were significantly associated with bipolar disorder. Among the clusters of combinations of 10 SNP genotypes one cluster was found to be significantly associated with bipolar disorder.

This cluster (Table 1 and S1 Table) contains 21 combinations which show large overlap with respect to SNP genotypes, so even though each combination contains 10 SNP genotypes the total number of SNP genotypes in the cluster is only 31.

It is noteworthy that only two of these 31 SNP genotypes are found in the other clusters, indicating that the cluster of combinations of 10 SNP genotypes represent a distinct genetic subgroup of bipolar patients.

It is also noteworthy that 28 of the 31 SNP genotypes are the normal homozygote, only three are the heterozygote and none is the variant homozygote. In the clusters of combinations of three and four SNP genotypes many of the genotypes were the heterozygote or the variant homozygote. This result suggests that small combinations of only three or four SNP genotypes may contribute to the risk of bipolar disorder when they contain relatively rare SNP genotypes, whereas combinations containing more common SNP genotypes need to be larger to constitute a risk factor. This suggestion is in line with the results from a study of oral cancer, where it was found that combinations of relatively few SNP genotypes taken from several genes belonging to the DNA repair system were a risk for oral cancer, whereas combinations where the genotypes were from only one gene belonging to the DNA repair system would have to contain relatively many SNP genotypes to constitute a risk factor [4].

We have not been able to find single SNP genotypes or single combinations of SNP genotypes significantly associated with bipolar disorder, but clusters of combinations have been found to be significantly associated with bipolar disorder. A cluster may thus be seen as a kind of a polygenic score, similarly to a polygenic score in a genome-wide association study, where a score based on genetic variants with low *p*-values may have higher predictive value than the single genetic variants. A cluster based on combinations found exclusively in patients (instead of variants with low *p*-values) may be significantly associated with a disorder in contrast to the combinations in the cluster. In that way the cluster can be seen as a risk factor for the disorder as such, but not as a risk factor for the single patient, as no patient probably will have all the combinations from the cluster in his genome. However, accumulation of combinations from the clusters in the genome of the single patient may be a personal risk factor.

Such a model may be true for many polygenic disorders, and the methods described in the present study facilitate analysis of combinations of genetic variants in data sets from older as well as new studies. Many genetic variants are known and for some polygenic disorders all variants behind the disorders may already have been found. However, the genetics of the disorders will remain unknown until the disease-causing combinations of these variants have been identified.

## Supporting information

S1 Table The table shows the 21 combinations of 10 SNP genotypes. After each combination is shown the genotypes, 0 normal homozygote, 1 heterozygote. The last column shows the dummy id for the patients having the combinations in their genome.
(XLSX)
